# Recognition of fold- and function-specific sites in the ligand-binding domain of the thyroid hormone receptor-like family

**DOI:** 10.3389/fendo.2022.981090

**Published:** 2022-09-29

**Authors:** Sonia Verma, Soumyananda Chakraborti, Om P. Singh, Veena Pande, Rajnikant Dixit, Amit V. Pandey, Kailash C. Pandey

**Affiliations:** ^1^ Parasite-Host Biology Group, ICMR-National Institute of Malaria Research, New Delhi, India; ^2^ Pediatric Endocrinology, Diabetology, and Metabolism, University Children’s Hospital, Bern, Switzerland; ^3^ Translational Hormone Research Cluster, Department of Biomedical Research, University of Bern, Bern, Switzerland; ^4^ Kumaun University, Nainital, Uttrakhand, India; ^5^ Academy of Scientific and Innovative Research, Ghaziabad, Uttar Pradesh, India

**Keywords:** nuclear receptor, thyroid hormone receptor-like family, ligand-binding domain, sequence conservation, phylogeny, relative entropy, multi-harmony, functional specificity

## Abstract

**Background:**

The thyroid hormone receptor-like (THR-like) family is the largest transcription factors family belonging to the nuclear receptor superfamily, which directly binds to DNA and regulates the gene expression and thereby controls various metabolic processes in a ligand-dependent manner. The THR-like family contains receptors THRs, RARs, VDR, PPARs, RORs, Rev-erbs, CAR, PXR, LXRs, and others. THR-like receptors are involved in many aspects of human health, including development, metabolism and homeostasis. Therefore, it is considered an important therapeutic target for various diseases such as osteoporosis, rickets, diabetes, etc.

**Methods:**

In this study, we have performed an extensive sequence and structure analysis of the ligand-binding domain (LBD) of the THR-like family spanning multiple taxa. We have use different computational tools (information-theoretic measures; relative entropy) to predict the key residues responsible for fold and functional specificity in the LBD of the THR-like family. The MSA of THR-like LBDs was further used as input in conservation studies and phylogenetic clustering studies.

**Results:**

Phylogenetic analysis of the LBD domain of THR-like proteins resulted in the clustering of eight subfamilies based on their sequence homology. The conservation analysis by relative entropy (RE) revealed that structurally important residues are conserved throughout the LBDs in the THR-like family. The multi-harmony conservation analysis further predicted specificity in determining residues in LBDs of THR-like subfamilies. Finally, fold and functional specificity determining residues (residues critical for ligand, DBD and coregulators binding) were mapped on the three-dimensional structure of thyroid hormone receptor protein. We then compiled a list of natural mutations in THR-like LBDs and mapped them along with fold and function-specific mutations. Some of the mutations were found to have a link with severe diseases like hypothyroidism, rickets, obesity, lipodystrophy, epilepsy, etc.

**Conclusion:**

Our study identifies fold and function-specific residues in THR-like LBDs. We believe that this study will be useful in exploring the role of these residues in the binding of different drugs, ligands, and protein-protein interaction among partner proteins. So this study might be helpful in the rational design of either ligands or receptors.

## Background

**Figure 1:**
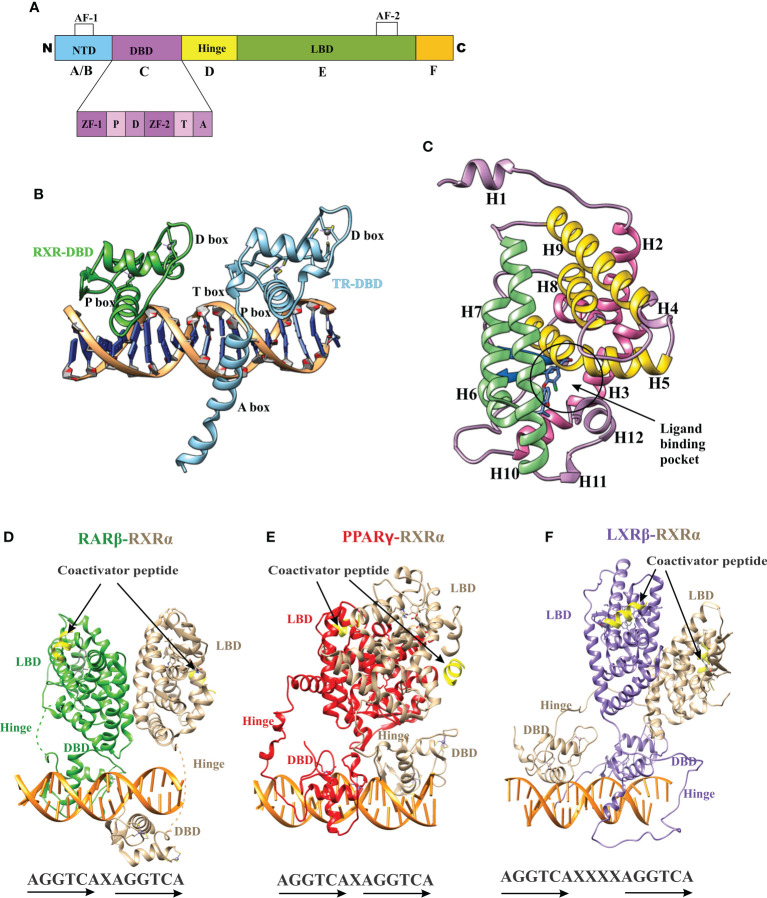
Domain structure of NRs. **(A)** The modular domain structure of NRs is composed of an unstructured NTD that contains the Activation Function 1 (AF-1) surface, a DBD that contains two highly conserved zinc finger motifs with P-, D-, T-, A- boxes (), a flexible hinge region, an LBD that binds to ligands and interacts with co-regulator proteins (Nuclear receptor co-activator 2 (NCOA2), Cyan) through the Activation Function 2 (AF-2) surface and less conserved F domain. **(B)** A cartoon structure of TR-RXR DBD dimer bound to its DNA response element showing various motifs of DBD. **(C)** The TR LBD structure shows its alpha-helical sandwich fold in which helix H5, H8, and H9 (Yellow) are sandwiched between helix H2, H3 (pink) on one side and by helix H6, H7, H10 (green) on the other side. The ligand binding pocket is shown at the bottom of the LBD. **(D)** The RAR-RXR heterodimer on its DNA response elements (direct repeats with single-base spacer.) **(E)** The PPAR-RXR heterodimer on its DNA response element (direct-repeats with single-base spacer). **(F)** The LXR-RXR heterodimer on its DNA response element (direct repeats with fur-base spacer). The Co-activator LXXLL motif is seen (yellow) in all structures on both LBDs.

The nuclear receptor (NR) superfamily is the most abundant transcription factor (TFs) of metazoans (1, 2). NRs play an essential role in many biological processes such as development, metabolism, physiology, reproduction, and inflammation, as well as in many pathological processes such as cancer, diabetes, rheumatoid arthritis, neurologic and psychiatric syndromes, immunosuppression, hormone resistance syndromes, cardiovascular diseases, metabolic syndrome, etc. (3–8). Most NRs share a similar structural architecture organized into five or six modular regions (A to F), sharing variable degrees of homology (9, 10) [**Figure 1A**]. A brief description of the NR domains is as follows. A/B: N-terminal domain (NTD): The NTD is a highly disordered domain with little sequence conservation. The NTD contains the activator function-1 region (AF-1), which interacts with a variety of co-regulator proteins in a cell and promoter specific manner and is subject to alternative splicing and post-translational modifications (10–12). C: DNA binding domain (DBD): The C region is the highly conserved DNA binding domain (DBD) responsible for recognition and binding to specific DNA sequences called hormone response elements (HREs) in the promoter region of a target gene. The DBD is composed of two tetracysteine zinc-finger motifs (the N-terminal motif (ZF-1) and C-terminal motif (ZF-2)) exclusive to NRs and other sequence elements known as P-, D-, T- and A- boxes (**Figure 1A**) (13). These structural elements contribute to HRE specificity (P-box), dimerization (D-box), and contact with the DNA backbone (T- and A-boxes) (13). In the RXR-TR DBD structure, the T- and A-boxes are shown to directly interact with the minor groove of the spacer element (i.e 4 bp in TR) (**Figure 1B**) (14). The T and A-boxes also responsible for assisting DBDs to conduct domain-domain interactions on DNA (15). The C-terminal region of DBD contains the nuclear localization signal and is located in the (16) D: Hinge Region: The hinge region is a variable-length and poorly conserved linker region between the DBD and the LBD. Variability in the length of this region allows the C and E domains to adopt different conformations and consequently contributes to the selection of the DNA binding sites. E: Ligand-binding domain (LBD): LBD is less conserved than DBD. The LBD is an allosteric signaling domain that binds to ligands and also interacts directly with DBD and co-regulator proteins in the multi-domain structure of the receptor organized on its DNA response element (**Figures 1D-F**) (17, 18). This domain commonly contains 12 α-helices and four β-strands that fold into three parallel layers to form an alpha-helical sandwich and a hydrophobic ligand-binding pocket (LBP) at the base of the receptor (**Figure 1C**). The C terminus of LBD contains another activation function surface (AF-2), which is composed of helices 3, 4, and 12. Helix 12, is conformationally dynamic and stabilized upon ligand binding, and appears to communicate with AF-2 to facilitate interaction with different co-regulator proteins (19). F: The F region is not present in all NRs and its function remains unclear.

According to sequence alignment and phylogenetic analyses of the conserved C and E domains, NRs were classified into eight subfamilies (NR1 to NR8) (**Table 1**) (9, 20), which share the conserved modular structure (**Figure 1A**), except NR7 members who have two DBDs (9). Non-canonical NRs lack one of the two conserved domains (C in vertebrates and E in invertebrates) and are grouped into the NR0 subfamily (9, 20, 21).

**Table 1:** The nuclear receptor superfamily is classified into eight families based on sequence conservation of the C (DBD) and E (LBD) domains.

S. No.	Family abbreviation	Family Name
1.	NR1	Thyroid Hormone Receptor-like
2.	NR2	Retinoid X Receptor-like
3.	NR3	Estrogen Receptor-like
4.	NR4	Nerve Growth Factor IB-like
5.	NR5	Steroidogenic Factor-like
6.	NR6	Germ Cell Nuclear Factor-like
7.	NR7	NRs with two DNA binding domains
8.	NR0	NRs lack either LBD or DBD

Data on the origin of nuclear receptors suggest that they were not hormonal receptors with a high affinity for a particular ligand, and that feature was acquired later during evolution (22). The THR-like family evolved as receptors with selective ligands (3, 22). The thyroid hormone receptor-like (THR-like) family is one of the largest families belonging to the nuclear receptor superfamily. THR-like receptors are ligand-activated transcription factors that bind to the DNA and regulate gene expression of related pathways and play an essential role in the development, metabolism, and physiology of an organism (**Table 2**) (2, 3, 23).

**Table 2:** Classification of THR-like receptors: The THR-like family is classified into 11 subfamilies, with functional details of the members and their corresponding ligands.

S. No.	Subfamily group	Subfamily Name	Subfamily Members	Ligands	Functional role of the receptor
1.	A	Thyroid hormone receptor	TRα (Thyroid hormone receptor alpha) TRβ (Thyroid hormone receptor beta)	Thyroid hormone	Regulation of metabolism, heart rate, and development of organisms
2.	B	Retinoic acid receptor	RARα (Retinoic acid receptor-α) RARβ (Retinoic acid receptor-β) RARγ (Retinoic acid receptor-γ)	Vitamin A and related compounds	Cell differentiation, cell proliferation, and apoptosis
3	C	Peroxisome proliferator-activated receptor	PPARα (Peroxisome proliferator-activated receptor-α) PPAR-β/δ (Peroxisome proliferator-activated receptor-β/δ) PPARγ (Peroxisome proliferator-activated receptor-γ)	Fatty acids, prostaglandins	Cellular differentiation, development, and metabolism (carbohydrate, lipid, protein) and tumorigenesis
4.	D	Rev-ErbA	Rev-ErbAα Rev-ErbAβ	Heme	Regulators of clock gene
5.	E	E78C-like (only found in arthropod, trematode, mollusk, nematode)	Eip78C (Ecdysone-induced protein 78C)	–	Development and regulate key processes during oogenesis. control ecdysone signaling in insects driving metamorphosis and molting
6.	F	RAR-related orphan receptor	RORα (RAR-related orphan receptor-α) RORβ (RAR-related orphan receptor-β) RORγ (RAR-related orphan receptor-γ)	Cholesterol, ATRA	Development of the cerebellum and lymph nodes, lipid metabolism, immune response, maintenance of bone. Circadian rhythm, bone metabolism, and retinal neurogenesis, Lymph node development and immune response, survival of T helper 17 cells
7.	G	CNR14-like (nematode)	Sex-1 (Steroid hormone receptor cnr14)	–	
8.	H	Liver X receptor-like	EcR (Ecdysone receptor, EcR (arthropod)) LXRβ (Liver X receptor-β) LXRα (Liver X receptor-α) FXR (Farnesoid X receptor) FXR-β (Farnesoid X receptor-β)	Ecdysteroids Oxysterols Bile acid Orphan	regulators of cholesterol, fatty acid, and glucose homeostasis
9.	I	Vitamin D receptor-like	VDR (vitamin D receptor) PXR (Pregnane X receptor) CAR (Constitutive androstane receptor)	Vitamin D Xenobiotics Androstane	Mineral Metabolism Detoxification Drug Metabolism and bilirubin clearance
10.	J	Hr96-like	Hr96/Daf-12 (Nuclear hormone receptor HR96)	Cholestrol/dafachronic acid	
11.	K	Hr1-like	Nuclear hormone receptor HR1	–	

Like nuclear receptors, THR-like receptors are modular in structure and have the following functional domains: (1) N-terminal domain (A/B), (2) DBD, (C) (3) flexible hinge region (D) (4) LBD, (E) (**Figure 1A**) (7). Among all the domains DBD is highly conserved, while the LBD is moderately conserved (3, 9, 23). The ligand-binding domain is the interaction site for ligands and co-regulators (co-activators (SRC1) or corepressors (NCoR, SMRT)) (24). Ligand binding in the binding pocket induces a conformational change in LBD, leading to the release of the co-repressors and the recruitment of co-activators. This conformational change in LBD propagates to the DNA binding domain through direct contact that promotes the DBD to bind the DNA and the subsequent transactivation of genes (9, 25). Structural findings in multiple nearly full-length complexes have now shown that DBD and LBDs communicate, through direct contacts (13, 15, 17) (**Figures 1D-F**). THR-like receptors form monomers, homodimers and heterodimers, most commonly with RXR on bipartite HREs composed of tandem AGGTCA ‘half site’. The HREs for RAR, PPAR, and LXR differ from one another only by the number of base pairs between two hexameric direct repeats (DR1-D5) (**Figures 1D-F**) (9). The LBD is the main interaction site for ligands, coactivators, and co-repressors and is also involved in the heteromerization of receptors. The ligands of THR-like receptors are small lipophilic molecules, including thyroid hormone, retinoic acid, fatty acids, heme, bile acids, and sterols. (**Table 2**). Based on sequence homology THR-like receptors are classified into 11 subfamilies i.e. (A) thyroid hormone receptor (THR) (B) retinoic acid receptor (RAR) (C) peroxisome proliferator-activated receptor (PPAR) (D) Rev-ErbA (E) E78C-like (F) RAR-related orphan receptor (ROR) (G) CNR14-like (H) Liver X receptor-like (I) Vitamin D receptor-like (J) Hr96-like (K) Hr1-like (**Table 2**) (20).

THR-like receptors represent novel targets for the development of therapeutic agents for the treatment of numerous diseases, including type 2 diabetes, obesity dyslipidemia, atherosclerosis, and metabolic syndrome (7). There have been many recent advances in the development of new therapeutic agents for a subset of these receptors, including peroxisome proliferator-activated receptors, liver X receptors, and farnesoid X receptors (4, 6, 7). Considering the therapeutic potentials, we analyze the LBD of the THR-like family to predict the fold and function-specific sites by using information-theoretic methods such as relative entropy and sequence harmony. In the present study, we perform extensive *in-silico* analysis to retrieve essential details regarding phylogenetic relationships and functionally important residues in the LBD of THR-like receptors. Taken together, the results provide a necessary framework for structural and functional studies of these important families of protein. This study would be helpful in detail in understanding the molecular basis of selective mutation, which might open new possibilities for the design of a new class of therapeutic agents.

## Materials and methods

### Data collection

**Figure 2:**
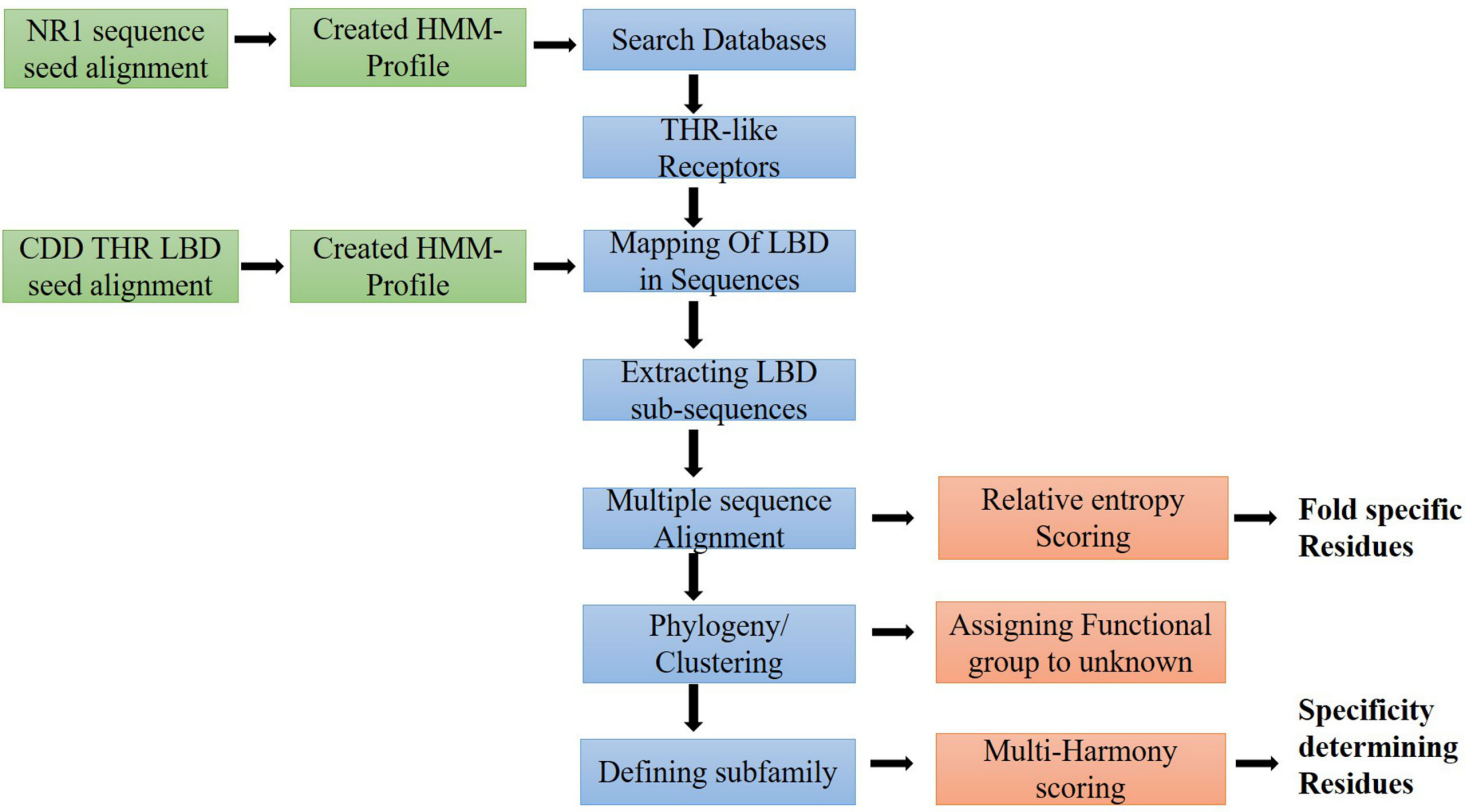
Workflow of the study: Flow diagram of the study to predict fold and function-specific residue *via* relative entropy and sequence harmony calculation.

To extract the protein sequences of the THR-like family, we searched various protein databases (NCBI, UniProt, EMBL) (https://www.ncbi.nlm.nih.gov/, https://www.uniprot.org/, https://www.embl.org/). We have created an HMM profile (Hidden Markov model) from the nuclear receptor family 1 (NR1) sequence seed alignment (26) (**Figure 2**). The HMM profile was searched against each protein database and full-length THR-like protein sequences were extracted using an internal script. CD-hit (Cluster Database at High Identity with Tolerance) was used to reduce sequence redundancy (27). Sequences that have more than 90% sequence similarities were removed from our database.

### Mapping of ligand-binding domain and sequence extraction

THR-like family proteins have mainly two conserved domains DBD and LBD. To extract the LBD sequences from the full-length THR-like protein, we need to map the start and end of the domain in the sequence. To map the LBD in the protein sequences, we have designed a strategy. The THR-like LBD sequence seed alignment was downloaded from the conserved domain database (CDD) (28). Seed alignment was used to create an HMM profile using HMMER 3.0 (26). The HMM profile was searched for in THR-like full-length sequences. The Hmmsearch (26) output provides the start and end of the LBD in each sequence. The LBD sequences were extracted by using an in-house script (**Figure 2**).

### Sequence and structure alignment

All THR-like LBD sequences were aligned using MAFFT with the default parameters (29) (**Figure 2**). In an evolutionary process, protein structures are more conserved than sequences. Therefore, to include the structure conservation information, we have created a structure sequence multiple sequence alignment of THR-like LBD. Structures of THR-like LBD protein belonging to different subfamilies (**Table S1**) were downloaded from the PDB database and aligned using the program STAMP (30), which is an integral tool in VMD (31). A structure-sequence MSA of THR-like LBD was created by aligning the structure profile and the sequence profile using the profile-profile alignment command in MAFFT (29). Jalview was to visualize and edit the MSA (32). The sequences of the CNR14-like subfamily (nematode), Hr96-like, and Hr1-like subfamily were removed from the analysis due to low significance (short length, lower number). Motif analysis was carried out by using the MEME (Multiple EM for Motif Elicitation) suite (33). MEME searches for repeated, ungapped sequence patterns that occur in DNA or protein sequences for motif prediction.

### Phylogenetic analysis of THR-like ligand-binding domain

A phylogenetic tree was generated from the alignment of multiple sequences of THR-like LBD using FastTree2 software with the default amino acid substitution model (34) (**Figure 2**). FastTree2 is known for its ability to handle large alignments and run speeds that are faster than other software. FastTree2 adds minimum-evolution subtree-pruning-regrafting (SPRs) and maximum-likelihood NNIs. FastTree2 uses heuristics to restrict the search for better trees and estimates a rate of evolution for each site. Further, the phylogenetic tree was visualized and analyzed by using Archaeopteryx software (35)

### Calculation of relative entropy (RE) scores of THR-like LBD

Relative Entropy (RE) scores were calculated by comparing the amino acid probability distribution for each column of the multiple sequence alignment with that of the background distribution.

RE or the Kullback–Leibler divergence (KL divergence) was originally introduced by Solomon Kullback and Richard Leibler in 1951 as the direct divergence between two distributions (Kullback and Leibler 1951). It is used to measure the difference between an amino acid distribution P and some background distribution P*_null_*. The RE score of column i is defined as


$$RE_{i}=\sum_{x=1}^{20}p_{i}(x)log\frac{p_{i}(x)}{p_{null}(x)}$$

Pi(x) is the probability of the occurrence of amino acid x in column ‘i’ of the MSA, where Pnull is the background probability of amino acid x, which is generally calculated as the probability of finding an amino acid x in all available protein sequences, i.e. protein sequences in Swiss-Prot database.

We used our in-house perl script to do the calculations (36, 37). The high-scoring residues were further mapped onto the thyroid hormone receptor alpha structure (PDB ID: 1NAV; Chain A) by carrying out profile-profile alignment between the sequence and structural alignment.

### Multi-harmony analysis

Many protein families have subfamilies with functional specialization, such as the binding of different ligands or the participation of different protein-protein interactions. A small number of amino acids generally determine functional specificity. Multiple sequence alignments are often used to reveal functionally important residues within a protein family and identification of key residues that determine functional differences between protein subfamilies. We have used Multi-Harmony, an interactive web server for detecting sub-type-specific sites in LBD of THR-like receptors (38). The multi-Harmony server uses a combined method of sequence Harmony (SH) (39) and multi-Relief (MR) (40) to detect subfamily-specific residues. Sequence Harmony is based on Shannon’s entropy and determines to what extent amino acid compositions between groups differ. Multi-Relief identifies residues based on RELIEF, a state-of-the-art machine learning technique for feature weighting.


$$SH_{i}^{A/B}=\sum_{x}p_{i,x}^{A}\log\frac{p_{i,x}^{A}}{p_{i,x}^{A}+p_{i,x}^{B}}$$

The SH score can be calculated as the relative entropy of group A relative to the sum of the probabilities of both groups ( *p* ^A^ + *p* ^B^).

### Mutation analysis

A collection of currently known natural-occurring THR-like LBD mutations was assembled From the UniProtKB database (41). The resulting dataset includes naturally occurring LBD mutations in the following receptors: PPAR, RAR, THR, LXR, VDR and ROR (**Table S2**). Based on the generated list, all mutations were classified according to our scoring scheme as fold and function-specific mutations. Furthermore, mutations were mapped to have an association with any disease in humans.

## Results

### Retrieval and downstream filtering of sequences

A total of 1276 THR-like receptor sequences were collected from the databases. We have used nuclear receptor family 1 (NR1) sequence seed alignment to search the database. Sequence redundancy was removed by CD-HIT software, keeping 90% sequence similarity as the clustering threshold. CD-hit provided 1176 sequence clusters. THR-like receptors consist of several domains out of them, two are major domains; DBD and LBD. As we were interested in studying LBD, we designed a strategy to fetch the LBD sequence out of a full-length THR-like sequence. We have created an HMM profile from the THR-like LBD sequence seed alignment downloaded from the conserved domain database. The HMM profile was searched against the THR-like full-length sequences database. We have used the bit score value (X=114) as the threshold per domain in the hmmsearch command against the THR-like sequence database. The hmmsearch output also provides the start and end of the LBD in each sequence. The LBD sequences were extracted by using an in-house script. The short length (between 100aa and 130aa) LBD domain sequences were removed, and finally, 1158 THR-like LBD sequences were used for the study.

### Multiple sequence alignment of THR-like LBD

**Figure 3:**
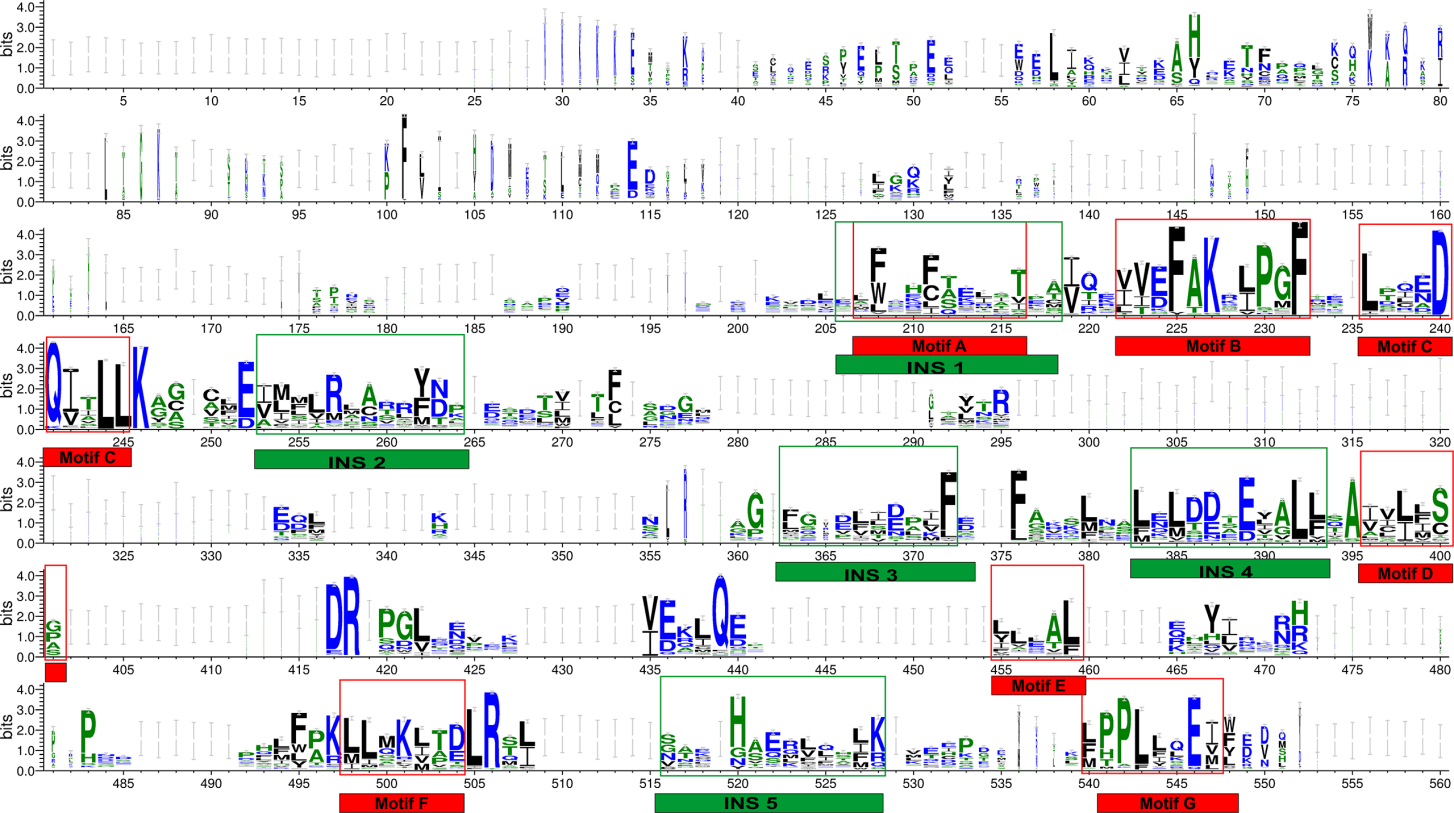
THR-like LBD sequence conservation logo: The sequence logo shows conservation patterns that emerged from the multiple-sequence alignment of THR-like LBDs. The overall height of the stack represents the conservation of the sequence at a particular position, and the frequency of the respective amino acid at that position is indicated by the height of the amino acid symbol. The conserved motifs (red boxes) and interaction sites (site for ligand, coactivators/corepressors and DBD binding) are highlighted (green boxes).

Multiple sequence alignment of THR-like LBD sequences was performed using MAFFT software. To check the accuracy of alignment, we performed a profile-profile alignment. As structures are more conserved than sequences, we aligned the THR-like LBD structure profile created using STAMP software (10 known structures of THR-like LBD proteins; details in **Table 2**) with the THR-like LBD sequence profile. We employed WebLogo 3 (http://Weblogo.threeplusone.com/create.cgi) (42) to visualize the conservation patterns that emerge from the profile-profile alignment. The WebLogo shown in **Figure 3** represents the conserved residues in the LBD region of the THR proteins. In the WebLogo plot, all the hydrophobic residues (YVMCLFIW) are colored black, while neutral amino acid (SGHTAP) and hydrophilic residues (RKDENQ) are colored green and blue, respectively (**Figure 3**). MEME Motif analysis provided 12 motifs in the protein sequences (**Figure 3**). These motifs of THR-like LBD provided information about seven highly conserved signaling motifs and five interaction sites. The interaction sites are the motifs where ligands, DBD and co-proteins interact. Motif A occupies consensus sequence positions 207-216. This is known as the inverse NR-box LLxxL motif and is one of the main ligand interaction sites. The NR box (NR interacting box LxxLL) plays an important role in the interaction of NRs with co-activators and therefore, in the regulation of transcription. Motif B occupies consensus sequence positions 222-232 and resides in a critical region for the coactivator function. Motif C also known as the LxxDDQ motif occupies positions 236-245 and is a region that is also critical to co-activator function. Motif D occupies positions 396-401 and plays a significant role in ligand binding. Motif E is an inverse NR-box (LLxxL) and occupies positions 455-459. Motif F occupies positions 498-504. Motif G occupies alignment positions 540-548. Motifs F and G are critical for coactivators binding.

### Phylogenetic analysis of THR-like LBD

**Figure 4:**
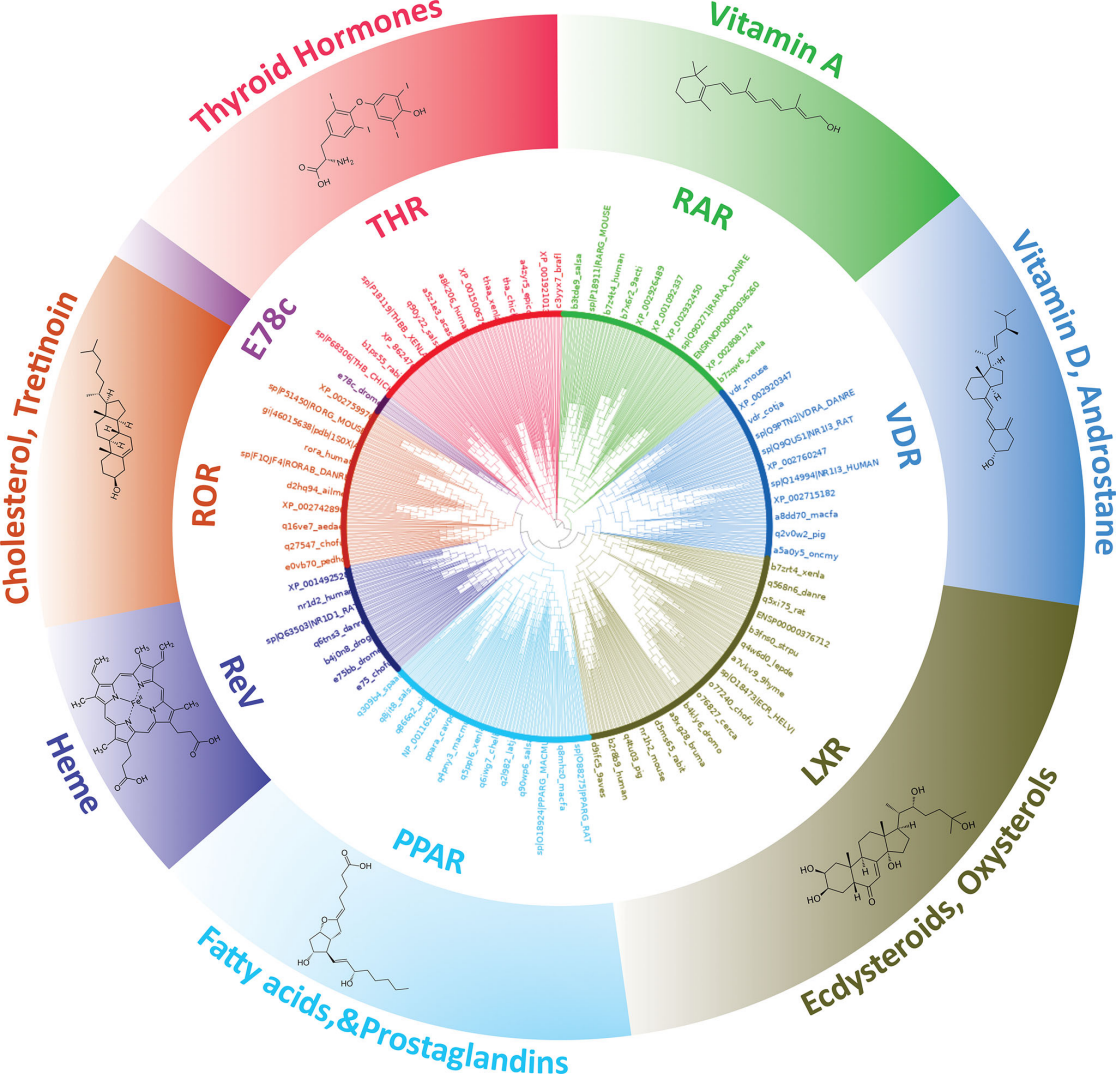
Phylogenetic tree of THR-like LBDs: Sequences are grouped into eight subfamilies: (Inner Circle receptors) THR (Red), RAR (Green), VDR (Blue), LXR (Olive green), PPAR (Cyan), ReV (Dark blue), ROR (Orange) and E78c (Purple). The ligands bind to the respective LBDs are shown on the outer circle.

THR-like family is the largest ligand-activated nuclear receptor family, with members existing only in metazoans and not found in plants, algae, fungi, and protists. Fasttree2 (34) which shows good and fast performance with large alignments was used for the tree construction from the THR-like LBD multiple sequence alignment. LBD sequences are clustered based on sequence homology and lineage. The resultant monophyletic tree showed clustering of the sequences into 8 subfamilies **(** **Figure 4**). The clustering of the branches is from points near the center of the tree. Cluster 1 includes all thyroid hormone receptors (red in the phylogenetic tree). The second cluster includes all members of the retinoic acid subfamily (green in the phylogenetic tree). The third group is Vitamin D receptor-like subfamily (colored blue). The fourth cluster is The Liver X receptor-like subfamily. The fifth cluster is the peroxisome proliferator-activated receptor subfamily. The sixth cluster is The Rev-ErbA subfamily. The seventh cluster is The RAR-related orphan receptor subfamily. The eighth cluster is The E78c subfamily from the phylogenetic tree analysis; it is predicted that there all these sequences are clustering based on their evolutionary relationships. Here is a brief description of the subfamilies.

Subfamily 1- Thyroid hormone receptor: Thyroid hormone receptors are activated by binding to thyroid hormone. Thyroid hormone receptors play a critical role in the regulation of metabolism, heart rate, and development of organisms (3). TH receptors also have non-genomic effects leading to second messenger activation, and the corresponding cellular response (43). Thyroid hormone receptors regulate gene expression by binding to hormone response elements (HREs) in DNA as monomers, homodimers, or heterodimers with other nuclear receptors (retinoid X receptor (RXR)). There are two main classes of the thyroid hormone receptor, alpha (THRA) and beta (THRB). Mutations in THRs are associated with thyroid hormone resistance disease (44). Clinically mutations have been observed in both the THR isoforms, however, THRB gene mutations are much more common.

Subfamily 2- Retinoic acid: Retinoic acid receptors (RAR) are activated by all-trans retinoic acid and 9-cis retinoic acid. There are three retinoic acid receptors, RAR-alpha, RAR-beta, and RAR-gamma. RARs form heterodimers with members of the retinoid X receptor (RXR) subfamily (25). RARs also have nongenomic effects *via* activating kinase signaling pathways, which fine-tune the transcription of the RA target genes. The disruption of RA signaling pathways is thought to underlie the etiology of several hematological and non-hematological malignancies, including leukemias, skin cancer, head/neck cancer, lung cancer, breast cancer, ovarian cancer, prostate cancer, renal cell carcinoma, pancreatic cancer, liver cancer, glioblastoma and neuroblastoma (45).

Subfamily 3- Vitamin D receptor-like: The VDR-like subfamily includes the vitamin D receptor, the pregnane X receptor, and the constitutive androstane receptor. Vitamin D receptor (VDR) heterodimerizes with retinoic acid upon binding calcitriol (an active form of vitamin D). The most potent natural agonist is calcitriol (1,25-dihydroxycholecalciferol) and the vitamin D2 homolog ercalcitriol, 1-alpha,25-dihydroergocalciferol) is also a strong activator (3). Mutations in the VDR gene are associated with type II vitamin D-resistant rickets. A disorder of vitamin D metabolism resulting in severe rickets, alopecia, secondary hyperparathyroidism and hypocalcemia. The pregnane X receptor (PXR), is a nuclear receptor that senses the presence of foreign toxic substances and in response up-regulates the expression of proteins involved in the detoxification and clearance of these substances from the body (46). PXR heterodimerizes with RXR and regulates the transcription of the cytochrome P450 gene CYP3A4. It is activated by a variety of compounds that induce CYP3A4, including dexamethasone and rifampicin (47). Constitutive androstane receptor (CAR) like PXR also functions as a sensor of endobiotic and xenobiotic substances. CAR and PXR play an important role in the detoxification of foreign substances and the metabolism of drugs. CAR-regulated genes are involved in drug metabolism and bilirubin clearance (48).

Subfamily 4- Liver X receptor-like: The liver X receptors (LXR) are closely related to nuclear receptors such as the PPARs, FXR, and RXR. Liver X receptors (LXRs) are important regulators of fatty acid, cholesterol, and glucose homeostasis. Two LXR have been identified as LXRα and LXRβ. LXRα and LXRβ form heterodimers with the obligate partner retinoid X receptor (RXR), which is activated by 9-cis-13,14-dihydroretinoic acid (49). The LXR/RXR heterodimer can be activated with an LXR agonist (oxysterols) or an RXR agonist (9-cis-13,14-dihydroretinoic acid). Oxysterols, oxygenated derivatives of cholesterol, such as 22(R)-hydroxycholesterol, 24(S)-hydroxycholesterol, 27-hydroxycholesterol, and cholesterol acid, are natural ligands for LXR (49).

Subfamily 5- Peroxisome Proliferator-activated receptor: Peroxisome proliferator-activated receptors (PPARs) play essential roles in the regulation of cellular differentiation, development and metabolism (carbohydrate, lipid, protein) and tumor genesis of higher organisms. PPARs have three isoforms termed alpha, gamma, and delta (beta). PPARs heterodimerize with the retinoid X receptor (RXR) and regulate the transcription of targeted genes. Endogenous ligands for the PPARs include free fatty acids, eicosanoids, and Vitamin B3 (50).

Subfamily 6-Rev-ErbA: The Rev-Erb proteins are members of the nuclear receptor (NR) superfamily of intracellular transcription factors and key regulatory components of the circadian clock. There are two forms of the receptor, Rev-Erb alpha and Rev-Erb beta. These receptors act as key regulators of clock gene expression through transcriptional repression of Bmal1. Through their regulation of clock-controlled genes, the Rev-Erb proteins affect several physiological processes throughout the body, including metabolic, endocrine, and immune pathways (51).

Subfamily 7-RAR-related orphan receptor: The RAR-related orphan receptors (RORs) are intracellular transcription factors. There are three isoforms of ROR, ROR-α, -β, and -γ. The RORs are somewhat unique in that they appear to bind as monomers to hormone response elements as compared to other nuclear receptors which bind as dimers. RORs are activated by oxysterols. Some other natural substances have also been reported to bind to RORs, such as all-trans-retinoic acid binds with high affinity to ROR-β and -γ but not ROR-α. RORs act as lipid sensors and hence may play a role in the regulation of lipid metabolism (52).

Subfamily 8- Ecdysone-induced E78c: The members of the 78c protein induced by ecdysone are found in arthropods, trematodes, mollusks, and nematodes. E78c protein has been linked to insect development and metamorphosis (53). Ecdysone-induced protein 78C (E78), a nuclear hormone receptor closely related to *Drosophila* E75 and mammalian Rev-Erb and Peroxisome Proliferator-Activated Receptors was originally identified as an early ecdysone target. The previous study shows that E78C plays a role in oogenesis and is important for proper egg production and maternal control of early embryogenesis (53).

### Prediction of fold-specific residues: Results of RE calculation

**Figure 5:**
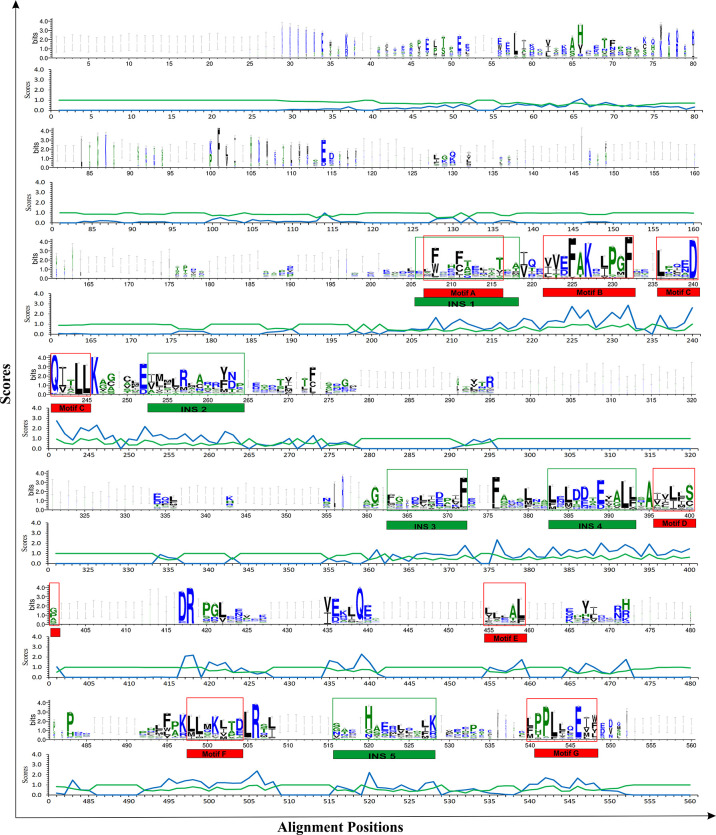
Mapping of RE (relative entropy, blue) and SH (sequence harmony, green) scores onto the sequence WebLogo of THR-like receptors LBDs. RE peeks (upward, blue) highlight the alignment position, which is structurally important (fold-specific), and SH peeks (Downward) show that the alignment is of functional significance (function-specific). Highlighted boxes represent the conserved motifs (red) and interaction sites (green).

**Figure 6:**
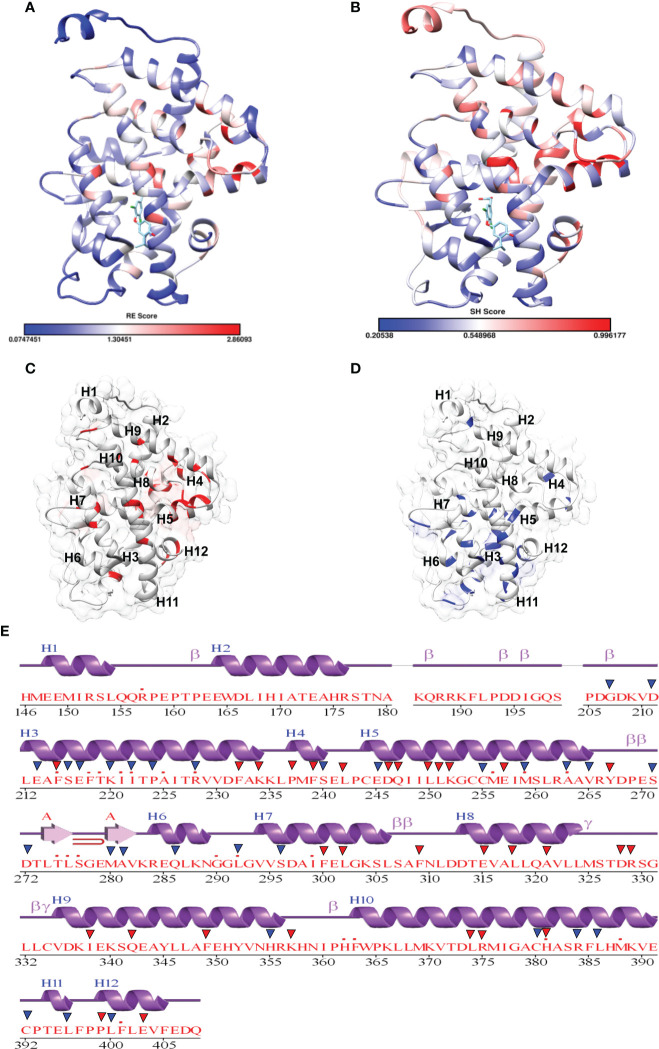
Mapping of scores in the THR LBD structure: **(A, B)** Mapping of the relative entropy score (RE) and sequence-harmony (SH) score onto the Human THR LBD structure (PDB, 1NAV), respectively (low score: Blue, High Score Red). **(C)** Highlighting High RE scoring residues onto the THR LBD (Red). **(D)** Highlighting low SH Scoring residues onto the THR LBD (Blue). **(E)** High RE (red triangle) and low SH (Blue triangle) scoring residue positions are marked on the secondary structure representation of THR LBD.

Fold-specific residues are responsible for maintaining the overall fold of the protein. Hence, such residues should be conserved across the THR protein alignment; an in-house Perl script was used to predict the fold-specific residues by employing a relative entropy calculation (RE). RE calculations identify residues whose probability distribution is significantly different from their background probability distribution. RE, therefore, predicts residues that are not identified by the traditional scoring methods (36). RE scores calculated for each MSA alignment position were plotted against sequence conservation in WebLogo of THR-like LBD. High RE scores are represented by a sharp upward peak (blue color) in the graph at an alignment position (**Figure 5**). It was observed that the residues identified as fold-specific are scattered across the helices (**Figures 6A, C**). Interestingly, H4, H6, H8, H9, H10, and H11 contain certain residues with very high RE scores. To gain insight into the distribution of the high-scoring residues on a 3D structure, the top 30 residues with the highest scores were mapped onto the crystal structure of the thyroid hormone receptor alpha of homo sapiens (PDB ID: 1NAV). **Table 3** summarizes the details of all residues which were indicated in the structure.

**Table 3:** Residues with high Relative entropy (RE) scores. Residues are classified as fold-specific residues.

S.No.	RE_score	Res name	Res Number
1.	2.86093	F	239
2.	2.736545	F	232
3.	2.735418	Q	247
4.	2.610201	D	246
5.	2.39529	K	234
6.	2.372856	R	375
7.	2.334283	L	302
8.	2.314478	K	252
9.	2.293988	Q	342
10.	2.222085	H	381
11.	2.191814	R	329
12.	2.183281	E	257
13.	2.148886	P	237
14.	2.095029	D	328
15.	2.073641	L	250
16.	2.023249	E	315
17.	1.922814	A	321
18.	1.875692	L	318
19.	1.870668	L	242
20.	1.77909	F	300
21.	1.773385	K	357
22.	1.748892	L	251
23.	1.747452	F	349
24.	1.702621	P	399
25.	1.7008	Y	267
26.	1.687514	L	374
27.	1.672857	E	403
28.	1.657511	I	338
29.	1.648088	F	215
30.	1.634578	F	309

### Prediction of function-specific residues: Results of multi-harmony calculation

Keeping in view the therapeutics of the THR-like family, we utilized another information-theoretic measure to explore distinct sequence signatures in the subfamilies. Though the proteins belonging to the THR-like family share the same protein fold, their ligand recognition and binding are different and very specific to each subfamily. It was of interest to explore the differentially conserved residues in one subfamily compared with another. The superposition of THR-like LBD structures reveals that the top part of the receptor is more or less similar compared to the bottom (which contains the LBP). This variability across THR-like receptors at the ligand-binding region allows THR-like receptors to recognize a diverse set of ligands. Therefore, to predict the specificity determining residues in the THR-like LBD we have used the multi-harmony web server. The Sequence Harmony (SH) and Multi-Relief (mR) methods in one web server allow simultaneous analysis and comparison of specificity residues. The SH scores calculated for each alignment position from the MSA were plotted against sequence conservation in weblogo of THR-like LBD (**Figure 5**, green color). The SH score lies between 0 and 1. The lower score shows the differential conservation of the residues at that alignment position. Refinements with low SH scores are the specificities that determine the residues. Low SH scores are represented by a downward peak (green color) in the graph at an alignment position **(** **Figure 5** **).** 30 residues with the minimum SH scores (considered 0.4 SH score as cutoff) are provided in **Table 4**. To get an idea of the distribution of the minimum SH scoring residues on a 3D structure, the top 30 residues with the minimum SH scores were mapped onto the homo sapiens crystal structure of the thyroid hormone receptor alpha (PDB ID: 1NAV) (**Figures 6B, D**). The Result shows that most of the minimum SH scoring residues are around the ligand-binding cavity (**Figure 6D**). **Table 4** summarizes the details of all residues which were indicated on the structure and a detailed list of residues with consensus sequences in all other subfamilies at that alignment position is provided in the supplementary information (**Table S2**).

**Table 4:** Residues with lower sequence harmony scores (SH). Residues are classified as function-specific residues.

S.No.	SH_score	Res name	Res Number
1.	0.20538	C	392
2.	0.240834	S	271
3.	0.258853	K	220
4.	0.26986	P	224
5.	0.29622	L	292
6.	0.300094	S	277
7.	0.301198	A	281
8.	0.30187	S	216
9.	0.307977	M	280
10.	0.309991	G	207
11.	0.313866	A	263
12.	0.314308	E	213
13.	0.316865	R	228
14.	0.330136	C	244
15.	0.332867	S	240
16.	0.333702	C	380
17.	0.334945	M	259
18.	0.336439	H	355
19.	0.341662	I	222
20.	0.350112	E	217
21.	0.351103	V	265
22.	0.354989	R	384
23.	0.359096	C	255
24.	0.360052	L	386
25.	0.36292	S	296
26.	0.366648	D	272
27.	0.366816	Q	286
28.	0.369598	L	396
29.	0.371785	F	401
30.	0.372781	D	211

### Mapping of LBD mutants as function and fold-specific

**Figure 7:**
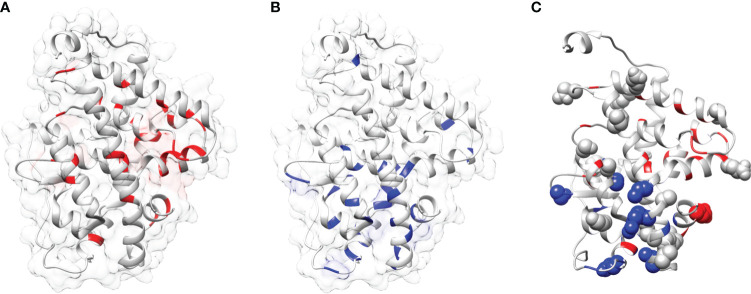
Mapping of mutations onto the THRA structure: **(A**, **B)** Mapping of fold-specific (red) and function-specific residues (blue) onto the human THRA LBD structure (PDB, 1NAV), respectively. **(C)** Mapping the mutation (sphere, gray) in THRA onto the structure shows that fold- and function specific sites are prone to mutations (spheres can be seen in blue and red regions).

**Figure 8:**
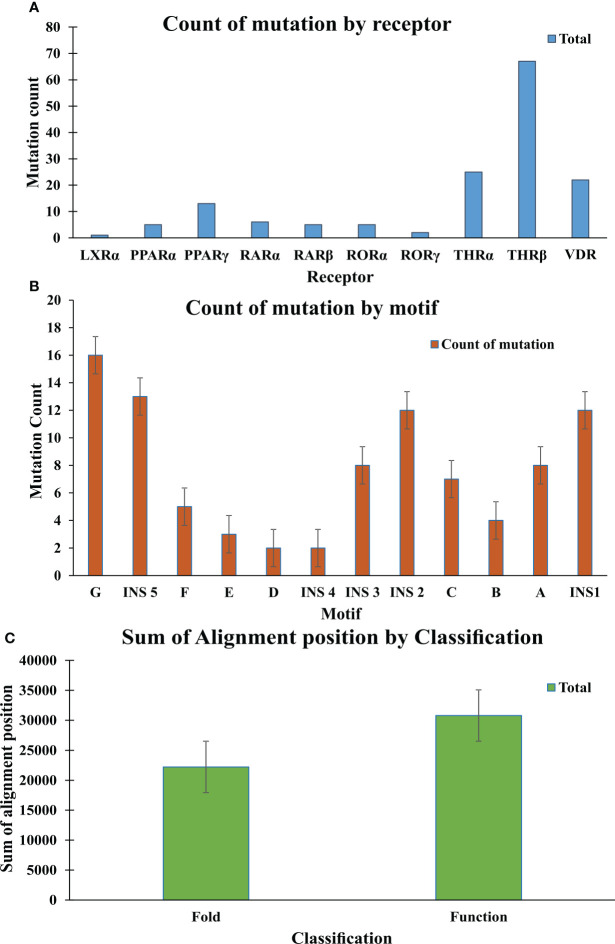
Mutation analysis graphs: **(A)** Graph shows the count of mutation by Receptor; THR-beta are more prone to mutation in comparison to other receptors **(B)** Graph shows the count of mutation by motifs. Motif G, INS 5, INS 2, INS1 are more susceptible to the mutation. **(C)** The graph shows the sum of the alignment positions classified as fold- and function-specific.

In this study a list of all known natural mutations similar to THR-like LBD was composed in this study from the UniProt knowledge database and the literature search (**Table S3**). Natural mutations were found in THRα, THRβ, RARα, RARβ, VDR, LXRα, PPARα, PPARγ and RORα, RORβ, and RORγ. Further mutations were classified according to our scoring scheme as fold and function specific (**Table S3**). THRA structure was used to map the predicted fold-, function-specific residues and natural mutations in THRA (**Figures 7A–C**). We have compiled a list of mutations that are associated with diseases in humans **(** **Tables 5** **,** **6** **)**. On the basis of mutation and the analysis of their results, it was observed that very highly conserved residues are not prone to mutations due to their essential role in protein function. The mutation in THRA LBD lies around the cavity **(** **Figure 7C** **)**. We have observed that receptor-wise, THRB has more mutation compared to THRA, VDR, and PPARA **(** **Figure 8A** **)**. Mutations are present in all motifs and interaction sites but residues lying at motif G, motif A, motif C, motif F, motif B and interaction sites INS 5, INS 1, INS 2, INS 3 are more prone to mutations **(** **Figure 8B** **)**. Mutational analysis also shows that most mutations are at the sites of functional significance compared to fold **(** **Figure 8C** **)**.

**Table 5:** THR-like receptors are involved in various diseases in humans.

S.No.	Receptor	Involvement in disease
1.	THRα	**Hypothyroidism, congenital, non-goitrous, 6 (CHNG6)** Disease description: A disease characterized by growth retardation, developmental retardation, skeletal dysplasia, borderline low thyroxine levels, and high triiodothyronine levels. There is differential sensitivity to the action of thyroid hormones, with retention of hormone responsiveness on the hypothalamic-pituitary axis and liver but skeletal, gastrointestinal, and myocardial resistance.
2.	THRβ	**Thyroid hormone resistance, generalized, autosomal dominant (GRTHD)** Disease description: An autosomal dominant disease characterized by high levels of circulating thyroid hormones (T3-T4), goiter, abnormal mental functions, increased susceptibility to infections, abnormal growth and bone maturation, tachycardia, and deafness. Affected individuals may also have attention deficit-hyperactivity disorders (ADHD) and language difficulties. Patients have normal or slightly elevated thyroid stimulating hormone (TSH). **Thyroid hormone resistance, generalized, autosomal recessive (GRTHR)** Disease description: An autosomal recessive disorder characterized by goiter, clinical euthyroidism, end-organ unresponsiveness to thyroid hormone, abnormal growth, bone maturation, and deafness. Patients also have high levels of circulating thyroid hormones, with elevated thyroid stimulating hormone. **Selective pituitary thyroid hormone resistance (PRTH)** Disease description: Variant form of thyroid hormone resistance and is characterized by clinical hyperthyroidism, with elevated free thyroid hormones but inappropriately normal serum TSH. Unlike GRTH, where the syndrome usually segregates with a dominant allele, the mode of inheritance in PRTH has not been established.
3.	RARα	Chromosomal aberrations involving RARA are commonly found in acute promyelocytic leukemia. Translocation t(11;17)(q32;q21) with ZBTB16/PLZF; translocation t(15;17)(q21;q21) with PML; translocation t(5;17)(q32;q11) with NPM. The PML-RARA oncoprotein requires both the PML ring structure and the coiled coil domain for both interactions with UBE2I, the location of nuclear microspeckles, and sumoylation. In addition, the coiled-coil domain functions in blocking RA-mediated transactivation and cell differentiation.
4.	RARβ	**Microphthalmia, syndromic, 12 (MCOPS12)** Disease description A form of microphthalmia, a disorder of eye formation that ranges from the small size of a single eye to the complete bilateral absence of ocular tissues (anophthalmia). In many cases, microphthalmia/anophthalmia occurs in association with syndromes that include non-ocular abnormalities. Patients with MCOPS12 exhibit variable features, including diaphragmatic hernia, pulmonary hypoplasia, and cardiac abnormalities.
5.	VDR	**Rickets vitamin D-dependent 2A (VDDR2A)** Description of the disease A disorder of vitamin D metabolism results in severe rickets, hypocalcemia, and secondary hyperparathyroidism. Most patients have total alopecia in addition to rickets.
6.	PPARγ	**Obesity** Description of the disease: A condition characterized by an increase of body weight beyond the limitation of skeletal and physical requirements as the result of excessive accumulation of body fat. **Lipodystrophy, familial partial, 3 (FPLD3)** Disease description: A form of lipodystrophy characterized by marked loss of subcutaneous fat from the extremities. Facial adipose tissue may be increased, decreased, or normal. Affected individuals show an increased preponderance of insulin resistance, diabetes mellitus, and dyslipidemia. PPARG defects can lead to type 2 insulin-resistant diabetes and hypertension. PPARG mutations may be associated with colon cancer. **Glioma 1 (GLM1)** Disease description Gliomas are benign or malignant central nervous system neoplasms derived from glial cells. They comprise multiforme astrocytomas and glioblastomas derived from astrocytes, oligodendrogliomas derived from oligodendrocytes, and ependymomas derived from ependymocytes. **Polymorphism** Genetic variations in PPARG define the quantitative trait locus 1 of the body mass index (BMIQ1). The body max index (BMI) reflects the amount of fat, lean mass, and body build. Genetic variations in PPARG influence the carotid intimal medial thickness (CIMT). CIMT is a measure of atherosclerosis that is independently associated with traditional atherosclerotic cardiovascular disease risk factors and coronary atherosclerotic burden. 35 to 45% of the variability in multivariate-adjusted CIMT is explained by genetic factors.
7.	RORα	**Intellectual developmental disorder with or without epilepsy or cerebellar ataxia (IDDECA)** Description of the disease An autosomal dominant neurodevelopmental disorder that manifests with variable features of mild to severe intellectual disability, developmental delay, autism spectrum disorder, cerebellar ataxia, and epilepsy.
8.	RORβ	**Epilepsy, generalized idiopathic 15 (EIG15)** Description of the disease An autosomal dominant form of generalized idiopathic epilepsy, a disorder characterized by recurring generalized seizures in the absence of detectable brain lesions and/or metabolic abnormalities. Generalized seizures arise diffusely and simultaneously from both hemispheres of the brain. Seizure types include juvenile myoclonic seizures, absence seizures, and generalized tonic-clonic seizures. EIG15 is characterized by the onset of variable types of seizures in the first decade of life.
9.	RORγ	**Disruption phenotype** Mice show decreased adipocyte size and high insulin sensitivity, leading to improved control of circulating fatty acids. Mutants are protected against hyperglycemia and insulin resistance in the state of obesity. Loss of circadian pattern of some clock genes expression in peripheral tissues and massive apoptosis of thymocytes. Knockout mice for isoform 2 lack all lymph nodes and Peyer patches, as well as LTi cells. They also show a reduction of T(H)17 cells in the lamina propria by at least 10 times to less than 1% of T (H) cells. Mice are less susceptible to autoimmune inflammatory diseases.

**Table 6:** List of THR-like receptor LBD mutations associated with diseases in humans.

S. No.	Receptor	Mutation	Classification	Alignment Position	Disease-Associated
1	THRα	N359Y	Function	484	in CHNG6; atypical phenotype; weak reduction in transcriptional activation
2	THRα	A263S	Function	258	in CHNG6; no effect on T3 binding; no effect on thyroid hormone-dependent transcriptional activation
3	THRβ	R429Q/W	Fold	506	in PRTH
4	THRβ	S350L	Function	368	in GRTHD
5	THRβ	V349M	Fold	367	in GRTHD
6	THRβ	V348E	Function	366	in GRTHD
7	THRβ	G347E/A	Fold	364	in GRTHD
8	THRβ	L346F	Fold/Function	363	in GRTHD
9	THRβ	G345R/V/S	Fold	361	in GRTHD
10	THRβ	G344E/A	Function	360	in GRTHD
11	THRβ	K342I	Function	343	in GRTHD
12	THRβ	L341P	Function	336	in GRTHD
13	THRβ	Q340H	Function	335	in GRTHD
14	THRβ	R338W/L	Fold	295	in GRTHD
15	THRβ	Del337T	Function	294	in GRTHD
16	THRβ	A335P	Function	292	in GRTHD
17	THRβ	E333D	Fold	277	in GRTHD
18	THRβ	G332R/E	Function	276	in GRTHD
19	THRβ	N331D	Function	275	in GRTHD
20	THRβ	L330S	Fold	273	in GRTHD
21	THRβ	T329I	Function	272	in GRTHD
22	THRβ	T327A	Function	269	in GRTHD
23	THRβ	Y321C/H	Fold	262	in GRTHD
24	THRβ	R320H/G	Function	261	in GRTHD
25	THRβ	A317T/S	Function	258	in GRTHD; impairs hormone binding
26	THRβ	R316H/C	Fold	257	in PRTH; impairs hormone binding
27	THRβ	A268G	Function	207	in GRTHD
28	THRβ	R243W	Fold	80	in GRTHD
29	THRβ	A234T	Function	71	in GRTHD; impairs hormone binding and ligand-dependent conformational changes
30	RARβ	R394C	Function	523	in MCOPS12; increased transcriptional response to retinoic acid ligands
31	RARβ	R394S	Function	523	in MCOPS12; increases transcriptional response to retinoic acid
32	RARβ	G303A	Fold	361	in MCOPS12; Increased transcriptional response to retinoic acid ligands
33	RARβ	L220P	Function	204	in MCOPS12; Increased transcriptional response to retinoic acid ligands
34	VDR	R391C	Fold	506	in VDDR2A
35	VDR	S360P	Function	456	in VDDR2A; loss of calcitriol receptor activity; loss of affinity for calcitriol; decreased ligand-independent localization to the nucleus; loss of interaction with RXRA; loss of interaction with NCOR1; loss of interaction with NCOA1; loss of sequence-specific DNA binding.
36	VDR	V346M	Fold	422	in VDDR2A; decreased calcitriol receptor activity; decreased affinity for calcitriol; decreased ligand-independent localization to the nucleus; loss of interaction with RXRA; decreased interaction with NCOR1; decreased interaction with NCOA1; decreased sequence-specific DNA-binding.
37	VDR	I314S	Fold	372	in VDDR2A
38	VDR	H305Q	Fold/Function	363	in VDDR2A; loss of calcitriol receptor activity; no effect on interaction with RXRA; changed interaction with NCOR1; loss of interaction with NCOA1; no effect on sequence-specific DNA binding.
39	VDR	R274L/H	Fold	257	in VDDR2A; loss of calcitriol receptor activity; decreased affinity for calcitriol by a factor of 1000; no effect on interaction with RXRA; changed interaction with NCOR1; loss of interaction with NCOA1; no effect on sequence-specific DNA binding.
40	PPARγ	P495L	Fold	542	in Diabetes
41	PPARγ	R425C	Fold	418	in FPLD3
42	PPARγ	F388L	Fold/Function	363	in FPLD3
43	PPARγ	V318M	Fold	216	in diabetes
44	PPARγ	R316H	Function	214	in colon cancer; sporadic; somatic mutation; partial loss of ligand-binding
45	PPARγ	Q314P	Function	212	in colon cancer; sporadic; somatic mutation; loss of ligand-binding
46	RORα	R462Q	Function	481	in IDDECA; loss of function in cerebellar development, when assayed in a heterologous system
47	RORα	S409R	Function	379	in IDDECA

## Discussion

In this study, we have combined both sequence and structural information from the THR-like LBDs into the multiple sequence alignment. Length−wise all THR-like ligand-binding domains are very similar. Finding conserved motifs should highlight regions of critical importance in ligand binding. Those regions can provide amino acid patterns that are a unique signature to the THR-like LBD. From the MSA we have found that the THR-like LBDs consist of seven conserved motifs and five interaction sites that are aligning with the previous studies (54). THR-like LBDs have the interaction site for the respective ligand and other co-proteins. It was observed that five interaction sites are present in the LBDs. Interaction site 1 lies within motif A, Interaction sites 2,3 and 4 lie between motifs C and D, and interaction site 4 lies between motifs F & G. The NR−boxes (LxxLL) or inverse NR−boxes (LLXXL) can bind to specific NR regions; they may have a role in the interaction between NRs, specifically in hetero and homo−dimerization.

The MSA was used to draw a phylogenetic tree of the THR-like LBDs. The phylogenetic analysis performed depicts the distinct separation of THR-like LBDs into the monophyletic branches. The THR-like LBDs are clustered according to the lineage.

Fold- and function-specific residues found in any protein of interest, and these residues are differentially conserved among the subfamilies and provide them functional specificity, such as binding at different ligands or being involved in different protein-protein interactions. To predict fold-specific and function-specific residues in the THR-like family, we used information-theoretic methods. A high RE score at the alignment position corresponds to the fold-specific residue, and on the other hand minimum, SH score at the alignment position in MSA corresponds to the function-specific site. The structure of the THR-like LBD is composed of 12 α-helices folded as an antiparallel tri-layer alpha-helical sandwich fold in which antiparallel alpha-helices (H5, H8, H9; the “sandwich filling”) are flanked by two alpha-helices on one side (H2, H3) and three on the other (H6, H7, and H10; the “bread”) (9). The ligand-binding cavity is within the interior of the LBD and just below three antiparallel alpha-helical sandwich “fillings” **(** **Figure 1C** **)**. The H12 is connected to H11 by a flexible loop which allows H12 to swing upon binding to a ligand, trapping the ligand and stabilizing the conformation of the NR (9). In this way, the LBD mediates recognition and interaction of ligands at the ligand-binding pocket (LBP), dimerization with LBDs from other NRs, interaction with co-regulators (coactivators [LxxLL motif in H4] or corepressors ([LxxxIxxxI/L motif in H1]) and ligand-dependent transactivation at the ligand-dependent activation function AF2 domain (H12), which enables the recruitment of co-regulators (21). The mapping of RE and SH scores on the human thyroid hormone structure has shown that high RE scoring residues are distributed onto the helixes in the LBD domain and are critical to maintaining the THR-like LBD fold, while the mapping of SH scores has shown that minimum SH scoring residues are distributed around the ligand bind pocket and contribute to ligand specificity in subfamilies (**Figure 6**). The dominance of nonpolar residues is more compared to polar and charged residues in both lists (fold-specific and function-specific residues) (**Table 3**, **4**). Additionally, to validate our study, we have compiled a list of natural mutations in THR-like LBDs and how they affect their function (**Table S3**). Since the interaction sites are mainly responsible for the ligand selectivity or co-proteins interactions, mutation analysis provides information that all the interaction sites and conserved motifs are prone to mutation (**Figure 8B**). Motif G and INS5 have high mutation count as compared to other motifs. Motif G and INS5 lie in helix H12 and H11, which play a crucial role in ligand trapping, binding, and stabilization of the receptors. Any mutation in these regions will lead to impairment of ligand binding, dimerization, and coreceptor binding. The selected mutations were further classified into fold- and function-specific residues according to our scoring scheme and analyzed how the mutation affects the function of the protein. Most of the mutations are function-specific which signifies that mutations are linked to impairment in ligand and co-receptor binding that leads to malfunctioning of the receptor and is associated with diseases in humans (55–60). We have found that 47 THR-like LBD mutations are critical and linked to diseases in humans (**Table 7**). Mutations in the LBD of THRα, THRβ, RARα, RARβ, VDR, PPARγ, RORα, RORβ, and RORγ receptors are associated with several diseases (**Table 6**). A brief description of the diseases is provided for reference (**Table 5**). Among all receptors, THRβ LBD is more prone to mutation and is involved in GRTHD, GRTHR, and PRTH diseases. THRβ mutations A317T/S and A234T are involved in GRTHD and affect hormone binding. THRβ mutation R316H/C is involved in PRTH and also impairs hormone binding (**Table 6**). THRβ residues R316 and A317 constitute the ligand binding domain. The A317 side chain interacts with iodine, sculpts the T3 in the ligand-binding pocket, and R316 is a part of the polar cluster in THRβ. Both mutants show decreased T3 affinity and weak transcription activity. The mutant R316H fails to dimerize which explains the weak transcription (61). The residue R316 forms two hydrogen bonds with the helix1, upon mutation to H316 both the hydrogen bond is lost which leads to displacement of helix1 and thereby loop after helix1. The stability of helix1 is required for ligand binding and dimerization (61). The mutant A317T repositions 3,5,3’-triiodothyroacetic acid, distending the face of the receptor that binds the coregulators and (61). Mutation analysis has also shown that some alignment positions are very critical (**Table 7**). Two and more than two receptors having mutations at these positions lead to impairment of LBD function resulting in the occurrence of disease. Three receptors have the mutation, THRβ (L346F), VDR (H305Q) and PPARγ (F388L) at alignment position 363 and are associated with GRTHD, VDDR2A and FPLD3 diseases, respectively (55, 57, 62). These findings suggest that the spatial position of the residues is also important with the residue (polar, nonpolar, and neutral) at that position and may help in the selectivity, binding and interaction of ligands with the coactivators and co-repressors.

**Table 7:** List of common THR-like receptors LBD mutations associated with diseases in Humans at an alignment position.

Receptor	Mutation	Classification	Alignment position	Disease-Associated
RARβ	R394C	Function	523	in MCOPS12; increased transcriptional response to retinoic acid ligands
RARβ	R394S	Function	523	in MCOPS12; increases transcriptional response to retinoic acid
THRβ	R429Q/W	Fold	506	in PRTH
VDR	R391C	Fold	506	in VDDR2A
THRβ	L346F	Fold/Function	363	in GRTHD
VDR	H305Q	Fold/Function	363	in VDDR2A; loss of calcitriol receptor activity; no effect on interaction with RXRA; changed interaction with NCOR1; loss of interaction with NCOA1; no effect on sequence-specific DNA binding.
PPARγ	F388L	Fold/Function	363	in FPLD3
THRβ	G345R/V/S	Fold	361	in GRTHD
RARβ	G303A	Fold	361	in MCOPS12; Increased transcriptional response to retinoic acid ligands
THRα	A263S	Function	258	in CHNG6; no effect on T3 binding; no effect on thyroid hormone-dependent transcriptional activation
THRβ	A317T/S	Function	258	in GRTHD; impairs hormone binding
THRβ	R316H/C	Fold	257	in PRTH; impairs hormone binding
VDR	R274L/H	Fold	257	in VDDR2A; loss of calcitriol receptor activity; decreased affinity for calcitriol by a factor of 1000; no effect on interaction with RXRA; changed interaction with NCOR1; loss of interaction with NCOA1; no effect on sequence-specific DNA binding.

## Conclusions

In conclusion, THR-like receptors are vital transcription regulators and therefore mentioned information acquired can be used in a variety of ways. Phylogenetic analysis also provided new insights for THR-like clustering and identified several key regions that exist through evolution in the NR LBD. The mutation analysis highlighted mutational hotspots while also providing insights into their effects, especially when they are localized in conserved motifs of THR-like LBD. Due to the diverse array of genes regulated by these proteins, along with the fact that many drugs are not explicitly specified for one receptor, drugs that target THR-like receptors tend to have unwanted side effects. Improving our understanding of THR-like receptors could pave the way for future therapeutics. Our study might be helpful to pharmaceutical companies and academic researchers in developing synthetic ligands that can specifically target these receptors. The conserved motifs and interaction sites can be used as selected targeted regions for rational design and discovery of novel drugs. The development of new drugs can be achieved through specific *in silico* techniques that can compose ligands, which can interact with specific conserved regions and force new alterations in the protein’s dynamics.

## Data availability statement

The original contributions presented in the study are included in the article/**Supplementary Material**. Further inquiries can be directed to the corresponding authors.

## Author contributions

All authors contributed to the implementation of the research. SV, KP, and AP conceived and designed the study. SV, SC, KP, AP, OS, VP, and RD wrote the paper. All authors contributed to the article and approved the submitted version.
