# Erratum: Recognition of fold- and function-specific sites in the ligand-binding domain of the thyroid hormone receptor-like family

**DOI:** 10.3389/fendo.2023.1175369

**Published:** 2023-03-10

**Authors:** Frontiers Production Office

**Affiliations:** Frontiers Media SA, Lausanne, Switzerland

**Keywords:** nuclear receptor, thyroid hormone receptor-like family, ligand-binding domain, sequence conservation, phylogeny, relative entropy, multi-harmony, functional specificity

An omission to the funding section of the original article was made in error. The following sentence has been added: “Open access funding was provided by the University of Bern”.

The original version of this article has been updated.

