# Supplementary material for: Recognition of fold- and function-specific sites in the ligand-binding domain of the thyroid hormone receptor-like family
Source: Front Endocrinol (Lausanne). 2022 Sep 29;13:981090. doi: 10.3389/fendo.2022.981090 (PMC9559826; doi:10.3389/fendo.2022.981090)
Supplement: Supplementary file 1 [file DataSheet_1.docx]

**Table S1:** List of 3D structures used in this study for structural alignment

| **PDB ID** | **Resolution** | **Structure description** | **DOI** |
| --- | --- | --- | --- |
| 2J14 | 2.80 Å | Peroxisome Proliferator-Activated Receptor Delta, Human | 10.1016/j.bmcl.2006.08.052 |
| 2NXX | 2.75 Å | Ecdysone Receptor, Tribolium Castaneum | 10.1038/sj.emboj.7601810 |
| 3BEJ | 1.90 Å | Structure Of Human FXR In Complex With MFA-1 And Co-Activator Peptide, Human | 10.1073/pnas.0710981105 |
| 1XV9 | 2.70 Å | Crystal Structure Of CAR/RXR Heterodimer Bound With SRC1 Peptide, Fatty Acid, And 5b-Pregnane-3,20-Dione, Human | 10.1016/j.molcel.2004.11.042 |
| 1FM6 | 2.10 Å | The 2.1 Å Resolution Crystal Structure Of The Heterodimer Of The Human Rxralpha And Ppargamma Ligand Binding Domains Respectively Bound With 9-Cis Retinoic Acid And Rosiglitazone And Co-Activator Peptides, Human | 10.1016/s1097-2765(00)80448-7 |
| 2HBH | 2.65 Å | Crystal structure of Vitamin D nuclear receptor ligand-binding domain bound to a locked side-chain analog of calcitriol and SRC-1 peptide, Danio rerio, Homo sapiens | 10.1016/j.abb.2007.01.031 |
| 3CQV | 1.90 Å | Crystal structure of Reverb beta in complex with heme Homo sapiens | 10.1371/journal.pbio.1000043 |
| 1XAP | 2.10 Å | Structure of the ligand-binding domain of the Retinoic Acid Receptor beta, Homo sapiens | 10.1038/sj.embor.7400235 |
| 1NAV | 2.50 Å | Thyroid Receptor Alpha in complex with an agonist selective for Thyroid Receptor Beta1 Homo sapiens | 10.1021/jm021080f |
| 1S0X | 2.20 Å | Crystal structure of the human RORalpha ligand-binding domain in complex with cholesterol sulfate at 2.2A Homo sapiens | 10.1074/jbc.M400302200 |

| **Table S2. List of function specific residues (with SH Score) in THRA and consensus sequence at the alignment position in other subfamilies** | | | | | | | | | | | |
| --- | --- | --- | --- | --- | --- | --- | --- | --- | --- | --- | --- |
| **S. No.** | **Alignment** | **PDB** | \| **ScoreSH** \| \| --- \| | \| **SF1(173)** \| \| --- \| | **SF2(158)** | **SF3(153)** | **SF4(234)** | **SF5(183)** | **SF6(101)** | **SF7(139)** | **SF8(17)** |
| 1 | 532 | CYS392 | 0.21 | Crs_ | Ifrlm_ | HPSafnglq_ | NDegks_ | Edkn_ | H-ape | Hqsvaft_y | W |
| 2 | 267 | SER271 | 0.24 | S_tgr | Q_stdnh | Tsekd | LST_amnpfdervi | Gv | IEcqsltd | N_trq | - |
| 3 | 213 | LYS220 | 0.26 | Kaq_re | E_d | De | Eknd_ | FC_gvys | QMacdfghinptvwyrselk | HIclqdve | Altm |
| 4 | 217 | PRO224 | 0.27 | P_ | Kaer | FYclmw | Lprm_nsvi | E_dvga | HP | Edngtaqh | P |
| 5 | 209 | SER216 | 0.3 | Skq_tg | D_hqse | Piqkaevmt | LARdeviyk_q | HYQ_fklnpr | QEgn_ | LQkge | Q |
| 6 | 275 | SER277 | 0.3 | SN_gf | S_it | Gdn | -LAfqsvg | Aps | Laimv | -nag | Dey |
| 7 | 292 | ALA281 | 0.3 | Akprt | T_ms | RKnylaqthcs | DPSlqfnverg_at | Fly | LTKVcsr | Y_fl | Yf |
| 8 | 363 | LEU292 | 0.3 | Lf | F_ | HFeilmny_ | IMLfv | Fli | ANcm | Clvdfaers | De |
| 9 | 198 | -- | 0.31 | Grv_dsn | Rgs_ | L-Trvsafdgpei | SEtiqkh_dgn | YSNkprgmead_qt | P-ta | Qy_nsk | Leiv |
| 10 | 206 | GLU213 | 0.31 | Elt_qd | G_eflvknd | SPegql_t | VEQpkdginstyar_ml | R_acesh | Ekrsq_ | Ewmqdav | W |
| 11 | 258 | ALA263 | 0.31 | A_iv | I_l | FSLca | MSTncgav | Lhistm | LF | M_l | Vai |
| 12 | 278 | MET280 | 0.31 | Mivl | L_m | -iqed | QFghmvky_elstar | Qhrtgksn | KQvyart | K_lvm | Atv |
| 13 | 221 | ARG228 | 0.32 | Rv_k | Krm | KQhdeng | ELrswyag_iv | Eskan | GEd | Yfmhsqn | R |
| 14 | 233 | SER240 | 0.33 | CSal_t | Tpqslav | Rmqvk | DQLmrcing_saet | SVlmrkdgqtia | QRcgntkas | M | Cvg |
| 15 | 238 | CYS244 | 0.33 | C_hyp | It | Ilvyptsam | Raclnpds_thq | Lsvm | Qkle | Qp | Q |
| 16 | 254 | MET259 | 0.33 | M_ | Lm | ICasml | M_il | Iacml | Lv | Vcla | W |
| 17 | 518 | CYS380 | 0.33 | Clray_ | K_ | AEli_q | HVQaityk_elmsn | Egqn_d | MLacdfghiknprstvwyeq_ | QRLhy_m | Kr |
| 18 | 215 | ILE222 | 0.34 | Iv_m | SA_c | SVNafklimt | TAvs_ | S_mlt | Fl | ILv | Vim |
| 19 | 470 | HIS355 | 0.34 | Y_ferh | RKst_n | GCfkthvleis_a | NItdrs_aml | ALgk_invts | QK_etr | K_eqvmsart | R |
| 20 | 210 | GLU217 | 0.35 | QEnp_h | K_hy | Hq | Hfgnly_rqi | GCR_qafs | Eahsd | DQLRfe | Q |
| 21 | 260 | VAL265 | 0.35 | V_iqsm | T_ilys | Tfipyrmeq | Rchlmkqe | Sap | CSmapg | Ra | Rk |
| 22 | 523 | ARG384 | 0.35 | Rvq_ | Rk_ | Qadhkner_ | QMat_lvi | Liqrvfh_m | Kr_qe | Key_sra | Hly |
| 23 | 250 | CYS255 | 0.36 | C_ | C | Amsclvt | TASv_k | Va | LTcv | CSa | F |
| 24 | 368 | SER296 | 0.36 | Sia | T_lms | Ltvm_i | Ilnstva | Mftli | VLitm | VIdl | Va |
| 25 | 525 | LEU386 | 0.36 | Lqvkm_ | Iflmv_ | -LQRshm | Fqyenrtlvs_im | Qe_h | VLm_ | Qhngilte_casm | De |
| 26 | 204 | ASP211 | 0.37 | Dkt_ | D_g | Vdglnkehras_pqi | ASReldvn_chpt | AE_krgs | G-alpsqv | MRactvphyq | Kr |
| 27 | 268 | ASP272 | 0.37 | E_qgkrd | D_ag | GMQksern | ED_alkrstcnmp | VLytm | RNdhksq | Ngqsedrh | TAhls |
| 28 | 335 | GLN286 | 0.37 | Qdh | Q_ | Dfhlqs | RDSaeiv_mpqtn | Fmv | SEDAhqvylt | AMVLft_ic | Q |
| 29 | 360 | GLY290 | 0.37 | G | A_gc | VAt_g | Aelq_fgts | K_sqr | GMacdefhiknpqrvwytls | Liqp_trs | Lmi |
| 30 | 378 | LYS304 | 0.37 | MKtlerv | N_akqsedg | YGVemdhriac_k | Rdnshk | Vesaitm | Esv | Kqrh | Nts |
| 31 | 539 | LEU396 | 0.37 | Lerd_ | Sdeqga_p | -nrsvtedqfk | Kevqr_ | Sfgphl_ca | -q | VLAHistre_ | Rk |
| 32 | 544 | PHE401 | 0.37 | F_ | Ivm_ | V-Liwm | Lf_ | L_ | R-hlmq | Yh_ | F |
| 33 | 200 | GLY207 | 0.38 | GDek_sn | Vsagi_ | -DLantsrpkge | REptnwhqyg_sd | Kghqidte_nr | -gptlvsnqh | RSN_alqk | E |

**Note:** SF stand for Subfamily. Amino acids in capital in the consensus sequences shows the highest conservation of that amino acid at that alignment position. Number in brackets (SF1 (176)) show the no of sequences in each subfamily.

| **Table S3. List of mutations located in the ligand binding domain of THR-like receptors** | | | | | | |
| --- | --- | --- | --- | --- | --- | --- |
| **S. No.** | **Receptor** | **Mutation** | **Classification** | **Alignment position** | **Motif** | **PMID** |
| 1 | THRα | E403K/X | Fold | 546 | G | 25670821 |
| 2 | THRα | P398R/S | Function | 541 | G | 10022432  25670821 |
| 3 | THRα | F397fs-406X | Fold | 540 | G | 27144938 |
| 4 | THRα | C392X | Function | 532 |  | 27144938 |
| 5 | THRα | V390A | Fold | 530 |  | 22507269 |
| 6 | THRα | M388I | Fold | 527 | INS 5 | 22507269 |
| 7 | THRα | R384H | Function | 523 | INS 5 | 27144938 |
| 8 | THRα | A382fs-388X | Function | 521 | INS 5 | 27144938 |
| 9 | THRα | C380fs-387X | Function | 518 | INS 5 | 27144938 |
| 10 | THRα | M369V | Function | 500 | F | 22507269 |
| 11 | THRα | N359Y | Function | 484 |  | 26303090 |
| 12 | THRα | Y352C | Fold | 467 |  | 22507269 |
| 13 | THRα | E350K | Fold | 465 |  | 22507269 |
| 14 | THRα | K337R | Function | 427 |  | 22507269 |
| 15 | THRα | S305P | Function | 379 |  | 22507269 |
| 16 | THRα | K288E/H | Function | 343 |  | 22507269 |
| 17 | THRα | S271I | Function | 267 |  | 22507269 |
| 18 | THRα | A264V | Fold | 259 | INS 2 | 22507269 |
| 19 | THRα | A263S | Function | 258 | INS 2 | 27144938 |
| 20 | THRα | E245V | Function | 239 | C | 22507269 |
| 21 | THRα | A225T/G | Function | 218 | INS 1 | 22507269 |
| 22 | THRα | E213D | Function | 206 | INS 1 | 22507269 |
| 23 | THRα | Q187X | Function | 78 |  | 22507269 |
| 24 | THRα | H184Q | Function | 75 |  | 22507269 |
| 25 | THRα | S183N | Function | 74 |  | 22507269 |
| 26 | THRβ | E460K | Function | 549 | G | 25905294 |
| 27 | THRβ | F459C/L | Function | 548 | G | 19268523  20237409 |
| 28 | THRβ | E457G | Fold | 546 | G | 24722129 |
| 29 | THRβ | L456S | Function | 545 | G | 22507269 |
| 30 | THRβ | F455S | Function | 544 | G | 19299458 |
| 31 | THRβ | L454fs-463, L454V | Fold | 543 | G | 17596672  8990194 |
| 32 | THRβ | P453H/A/L/T/S | Fold | 542 | G | 2153155,  8040303,  19268523,  18561095 |
| 33 | THRβ | P452ins, P452R | Fold | 541 | G | 8040303  24722129 |
| 34 | THRβ | F451S/I/L | Function | 540 | G | 27034829 |
| 35 | THRβ | P447T | Function | 533 |  | 19268523 |
| 36 | THRβ | C446R | Function | 532 |  | 22507269  8175986 |
| 37 | THRβ | K443E | Fold | 528 | INS 5 | 1587388 |
| 38 | THRβ | M442V/T | Fold | 527 | INS 5 | 1661299  19378427 |
| 39 | THRβ | L440P | Function | 525 | INS 5 | 19378427 |
| 40 | THRβ | R438H/C/P | Function | 523 | INS 5 | 8040303  30027432 |
| 41 | THRβ | H435L/Q/Y/R | Fold | 520 | INS 5 | 11889175  11701737 |
| 42 | THRβ | I431T/M | Fold | 508 |  | 11889175  19268523 |
| 43 | THRβ | R429Q/W | Fold | 506 |  | 8040303  12006711 |
| 44 | THRβ | D427G | Function | 504 | F | 22507269 |
| 45 | THRβ | T426I | Function | 503 | F | 10660344 |
| 46 | THRβ | F417L | Function | 494 |  | 22507269 |
| 47 | THRβ | H412R | Fold | 483 |  | 22507269 |
| 48 | THRβ | K411E | Fold | 472 |  | 22507269 |
| 49 | THRβ | F403L | Fold | 459 | E | 22507269 |
| 50 | THRβ | A387P | Function | 423 |  | 22507269 |
| 51 | THRβ | S380F | Fold | 400 | D | 22507269 |
| 52 | THRβ | M379T | Function | 399 | D | 22507269 |
| 53 | THRβ | L373P | Function | 393 | INS 4 | 22507269 |
| 54 | THRβ | S350L | Function | 368 | INS 3 | 24906004 |
| 55 | THRβ | V349M | Fold | 367 | INS 3 | 18363280 |
| 56 | THRβ | V348E | Function | 366 | INS 3 | 8889584 |
| 57 | THRβ | G347E/A | Fold | 364 | INS 3 | 1661299  17827792 |
| 58 | THRβ | L346F | Fold | 363 | INS 3 | 19268523 |
| 59 | THRβ | G345R/V/S | Fold | 361 |  | 25905294 |
| 60 | THRβ | G344E/A | Function | 360 |  | 19435825  21795843 |
| 61 | THRβ | K342I | Function | 343 |  | 15886199 |
| 62 | THRβ | L341P | Function | 336 |  | 19268523 |
| 63 | THRβ | Q340H | Function | 335 |  | 23806029 |
| 64 | THRβ | R338W/L | Fold | 295 |  | 8514853  8040303 |
| 65 | THRβ | Del337T | Function | 294 |  | 1653889 |
| 66 | THRβ | A335P | Function | 292 |  | 19268523 |
| 67 | THRβ | E333D | Fold | 277 |  | 17177139 |
| 68 | THRβ | G332R/E | Function | 276 |  | 8040303 |
| 69 | THRβ | N331D | Function | 275 |  | 19268523 |
| 70 | THRβ | L330S | Fold | 273 |  | 10724359 |
| 71 | THRβ | T329I | Function | 272 |  | 19820907 |
| 72 | THRβ | T327A | Function | 269 |  | 19378427 |
| 73 | THRβ | Y321C/H | Fold | 262 | INS 2 | 11756220  8040303 |
| 74 | THRβ | R320H/G | Function | 261 | INS 2 | 1314846  30027432 |
| 75 | THRβ | A318D | Fold | 259 | INS 2 | 11889175 |
| 76 | THRβ | A317T/S | Function | 258 | INS 2 | 25738994  8889584 |
| 77 | THRβ | R316H/C | Fold | 257 | INS 2 | 8381821  22319036 |
| 78 | THRβ | M313T/V | Function | 254 | INS 2 | 19268523  30027432 |
| 79 | THRβ | E299K | Function | 239 | C | 22507269 |
| 80 | THRβ | K289M | Function | 228 | B | 22507269 |
| 81 | THRβ | I280S | Fold | 219 |  | 22319036 |
| 82 | THRβ | A279E | Function | 218 | INS 1 | 19378427 |
| 83 | THRβ | I276L | Function | 215 | A, INS 1 | 21795843 |
| 84 | THRβ | T273A | Function | 212 | A, INS 1 | 22507269 |
| 85 | THRβ | A268G | Function | 207 | A, INS 1 | 19268523 |
| 86 | THRβ | V264D | Function | 203 |  | 9092799 |
| 87 | THRβ | Q252R | Function | 130 |  | 11756220 |
| 88 | THRβ | I250T | Function | 128 |  | 19378427 |
| 89 | THRβ | R243W | Fold | 80 |  | 9141558 |
| 90 | THRβ | Q235X | Function | 72 |  | 22507269 |
| 91 | THRβ | A234T | Function | 71 |  | 26273722 |
| 92 | THRβ | W219L | Function | 54 |  | 27034829 |
| 93 | RARα | M413T | Function | 547 | G | 9694705 |
| 94 | RARα | Q411X | Function | 545 | G | 1327285 |
| 95 | RARα | R394W | Function | 523 | INS 5 | 9694705 |
| 96 | RARα | M297L | Function | 336 |  | 9657734 |
| 97 | RARα | L290V | Function | 278 |  | 9694705 |
| 98 | RARα | R272Q | Fold | 257 | INS 2 | 9657734 |
| 99 | RARβ | I410S fs*15 | Function | 544 | G | 24075189 |
| 100 | RARβ | R394C | Function | 523 | INS 5 | 24075189 |
| 101 | RARβ | R394S | Function | 523 | INS 5 | 24075189 |
| 102 | RARβ | G303A | Function | 361 |  | 27120018 |
| 103 | RARβ | L220P | Function | 204 |  | 27120018 |
| 104 | VDR | R391C | Fold | 506 |  | 24246681 |
| 105 | VDR | T362I | Fold | 458 | E | 24246681 |
| 106 | VDR | S360P | Function | 456 | E | 19169476 |
| 107 | VDR | V346M | Fold | 422 |  | 12468277 |
| 108 | VDR | E329K | Fold | 389 | INS 4 | 8961271 |
| 109 | VDR | G319V | Function | 379 |  | 24246681 |
| 110 | VDR | Q317X | Function | 377 |  | 24246681 |
| 111 | VDR | I314S | Fold | 372 | INS 3 | 8961271 |
| 112 | VDR | H305Q | Fold | 363 | INS 3 | 24246681 |
| 113 | VDR | Y295X | Fold | 293 |  | 24246681 |
| 114 | VDR | W286R | Fold | 270 |  | 24246681 |
| 115 | VDR | R274L/H | Fold | 257 | INS 2 | 24246681 |
| 116 | VDR | I268T | Function | 251 | INS 2 | 24246681 |
| 117 | VDR | L263R | Function | 245 | C | 24246681 |
| 118 | VDR | Q259P/E | Fold | 241 | C | 19169476  24246681 |
| 119 | VDR | F251C | Fold | 232 | B | 24246681 |
| 120 | VDR | K246(3bp deletion) | Fold | 227 | B | 24246681 |
| 121 | VDR | L230V | Fold | 208 | A, INS 1 | 27114920 |
| 122 | VDR | L227P | Fold | 211 | A, INS 1 | 24246681 |
| 123 | VDR | R158C | Function | 136 |  | 24246681 |
| 124 | VDR | Q152X | Function | 130 |  | 24246681 |
| 125 | VDR | T146I | Function | 119 |  | 24246681 |
| 126 | LXRα | R415Q | Fold | 501 | F | 27253448 |
| 127 | PPARα | V227A | Function | 79 |  | 16288935 |
| 128 | PPARα | A268V | Function | 203 |  | 7684926 |
| 129 | PPARα | D304N | Fold | 240 | C | 18541586 |
| 130 | PPARα | G395R | Function | 427 |  | 20414453 |
| 131 | PPARα | R409T | Fold | 465 |  | 18541586 |
| 132 | PPARγ | P495L | Fold | 542 | G | 28208577 |
| 133 | PPARγ | H477L | Fold | 520 | INS 5 | 26756202 |
| 134 | PPARγ | R425C | Fold | 418 |  | 28208577 |
| 135 | PPARγ | D424N | Fold | 417 |  | 17766367 |
| 136 | PPARγ | F388L | Function | 363 | INS 3 | 25004973 |
| 137 | PPARγ | R385A/X | Function | 357 |  | 28208577  18713822 |
| 138 | PPARγ | Y355X | Function | 255 | INS 2 | 6412238 |
| 139 | PPARγ | K347X | Fold | 246 | C | 10394368 |
| 140 | PPARγ | L339X | Function | 238 | C | 18713822 |
| 141 | PPARγ | V318M | Fold | 216 | A, INS 1 | 23393388 |
| 142 | PPARγ | R316H | Function | 214 | A, INS 1 | 28208577 |
| 143 | PPARγ | Q314P | Function | 212 | A, INS 1 | 28208577 |
| 144 | PPARγ | R308P | Function | 206 | INS 1 | 29622583 |
| 145 | RORα | T476A | Function | 504 | F | 29656859 |
| 146 | RORα | R462Q | Function | 481 |  | 29656859 |
| 147 | RORα | S409R | Function | 379 |  | 29656859 |
| 148 | RORα | R340Pfs*17 | Function | 228 | B | 29656859 |
| 149 | RORα | Q315Lfs*51 | Function | 203 |  | 29656859 |
| 150 | RORγ | Q441X | Fold | 439 |  | 26160376 |
| 151 | RORγ | Q329X | Fold | 220 |  | 26160376 |
